# “Don’t Phos Over Tau”: recent developments in clinical biomarkers and therapies targeting tau phosphorylation in Alzheimer’s disease and other tauopathies

**DOI:** 10.1186/s13024-021-00460-5

**Published:** 2021-06-05

**Authors:** Yuxing Xia, Stefan Prokop, Benoit I. Giasson

**Affiliations:** 1 Department of Neuroscience, College of Medicine, University of Florida, BMS J483/CTRND, 1275 Center Drive, Gainesville, FL 32610 USA; 2 Center for Translational Research in Neurodegenerative Disease, College of Medicine, University of Florida, Gainesville, Florida 32610 USA; 3 Department of Pathology, College of Medicine, University of Florida, Gainesville, Florida 32610 USA; 4 McKnight Brain Institute, College of Medicine, University of Florida, Gainesville, Florida 32610 USA

**Keywords:** Alzheimer’s disease, Frontotemporal lobar degeneration, Tauopathy, Tau phosphorylation, Cerebrospinal fluid, Plasma, Tau immunotherapy, Kinase inhibitor, Phosphatase activator

## Abstract

Phosphorylation is one of the most prevalent post-translational modifications found in aggregated tau isolated from Alzheimer’s disease (AD) patient brains. In tauopathies like AD, increased phosphorylation or hyperphosphorylation can contribute to microtubule dysfunction and is associated with tau aggregation. In this review, we provide an overview of the structure and functions of tau protein as well as the physiologic roles of tau phosphorylation. We also extensively survey tau phosphorylation sites identified in brain tissue and cerebrospinal fluid from AD patients compared to age-matched healthy controls, which may serve as disease-specific biomarkers. Recently, new assays have been developed to measure minute amounts of specific forms of phosphorylated tau in both cerebrospinal fluid and plasma, which could potentially be useful for aiding clinical diagnosis and monitoring disease progression. Additionally, multiple therapies targeting phosphorylated tau are in various stages of clinical trials including kinase inhibitors, phosphatase activators, and tau immunotherapy. With promising early results, therapies that target phosphorylated tau  could be useful at slowing tau hyperphosphorylation and aggregation in AD and other tauopathies.

## Main text

### Overview of tau structure and function

**Fig. 1:**
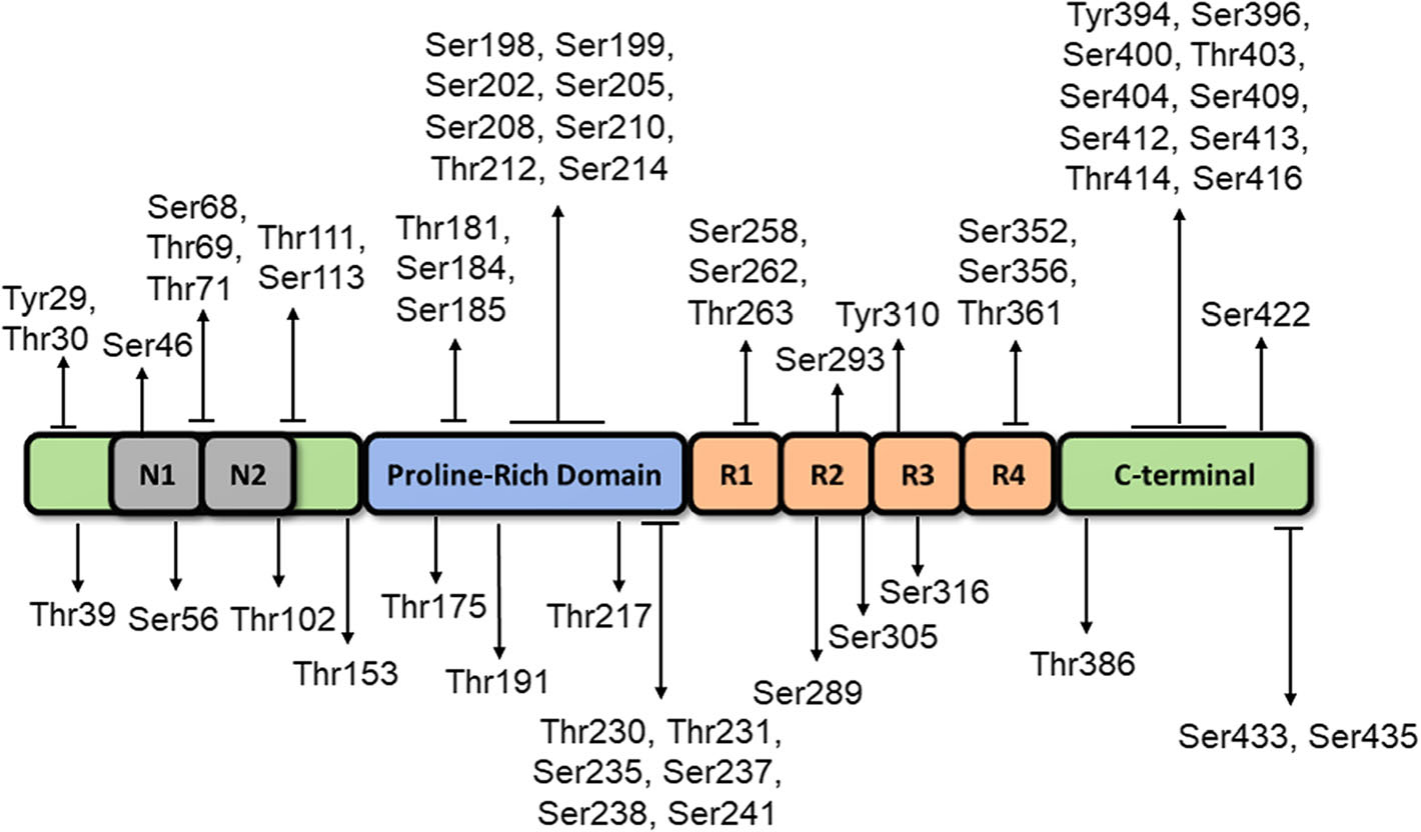
Schematic showing 2N4R tau (441 amino acids), the longest isoform expressed in human brain. Tau protein contains major structural domains including N-terminal domain with N1 and N2 inserts, proline rich region, four major microtubule-binding repeats (R1-R4), and C-terminal domain. The N1, N2 and R2 regions can be alternatively spliced in the human brain resulting in 6 isoforms: 0N3R, 1N3R, 2N3R, 0N4R, 1N4R, and 2N4R. The position of identified phosphorylation sites found in AD brains are shown [1–5]

Tau protein is encoded in the microtubule associated protein tau (*MAPT*) gene on chromosome 17. In the human brain, alternative RNA splicing of exons 2, 3, and 10 lead to the expression of six major tau isoforms: 0N3R, 1N3R, 2N3R, 0N4R, 1N4R, and 2N4R (Fig. 1) [6–8]. Exon 2 and 3 encode two different N-terminal domains of 29 amino acids and their presence or absence results in either 0 N, 1 N, or 2 N isoforms. Exon 10 encodes the second microtubule-associated binding repeat (MTBR) and its alternative splicing differentiates between 3R and 4R tau isoforms [9]. 3R tau isoforms contain the first, third, and fourth MTBR, while 4R tau isoforms contain all four MTBR. In the adult human brain, the six major tau isoforms are expressed at different abundance: 0 N isoforms make up ~ 40% of all isoforms, while ~ 50% are 1 N isoforms and ~ 10% are 2 N isoforms [10, 11]. Expression of 3R and 4R isoforms is relatively equal throughout the brain [10, 11].

Tau is expressed and localized primarily in the axons of neurons and at lower levels in glial cells [12, 13]. Tau is overall hydrophilic and basic, consisting of several structural regions including the N-terminal projection region, proline-rich domain, MTBR, and the C-terminal region [14] (shown in Fig. 1). The MTBR region is positively charged facilitating binding with negatively charged microtubules (MT) [15, 16]. The proline rich domain is also involved in MT binding and regulation; its partial or complete deletion can lead to MT impairment [14].

Since its discovery in 1975, tau was named as a tubulin associated unit due to its functions in promoting the growth and extension of MT [17]. The MTBR within tau have a high homology with other microtubule-associated proteins (MAPs) that maintain MT. Tau can bind to the interface between alpha-tubulin and beta-tubulin heterodimer and stabilize the MT structure [18–20]. Recently, it has been proposed that the effects of tau may be more indirect, and tau elongates the labile domain rather than stabilize MT [21]. Nonetheless, tau is important for the homeostatic growing and shrinking of MT along the neuronal axon. Tau can also regulate motor proteins such as dynein and kinesin and affect the rates of axonal transport [22, 23].

Tau is involved in neuronal maturation and maintenance of cytoarchitecture, evidenced by smaller axons and dendrites in tau knockout mice [24, 25]. Some aged tau knockout mice developed spatial memory deficits [26], while another lineage of tau null mice developed motor deficits without cognitive changes [27]. Differences in genetic background may explain the divergence in behavioral phenotypes [28]. Interestingly, tau knockdown by AAV-delivered RNA interference in adult mice impaired spatial learning and memory and lead to decreased synaptic proteins and dendritic spine density [29], while restoration of tau expression rescued the behavioral deficits in these mice. Overall, these studies suggest that loss of tau function is likely detrimental for cognition and memory.

### Physiologic roles of tau phosphorylation

Tau protein can be post-translationally modified by enzymatic additions of acetylation, methylation, glycosylation, ubiquitination, and many others [30, 31]. As discussed below, of these post-translation modifications (PTMs), phosphorylation is one of the earliest and most prevalent modifications associated the formation of pathological inclusions. In the longest tau isoform expressed in the human CNS (2N4R isoform), there are over 85 potential phosphorylation sites represented by either serine, threonine, or tyrosine residues [8]. Phosphorylation is performed by kinases that add a phosphate group from adenosine triphosphate. Three major classes of kinases can phosphorylate tau [32]: proline-directed kinases such as glycogen synthase kinase 3 beta (GSK-3β) or cyclin dependent kinase 5 (CDK5) [33, 34], non-proline-directed kinases such as tau-tubulin kinases (TTBK) or microtubule affinity regulated kinases (MARK) [35, 36], and tyrosine kinases such as Fyn or Abl kinases [37, 38]. Dephosphorylation or the removal of a phosphate group is performed by phosphatases. Protein phosphatase 2A (PP2A) is the major enzyme that accounts for ~ 71% of the total tau dephosphorylation activity [39, 40], while other phosphatases involved include PP1, PP5, and PP2B [40].

Tau phosphorylation is elevated during some physiologic processes such as development, hibernation, and hypothermia. During development, fetal tau is highly phosphorylated at multiple sites including Thr181, Ser199, Ser202, Thr205, Thr231, Ser396 and Ser404 [41–43]. After the developmental period, tau phosphorylation returns to steady state levels in part because mature brains have higher phosphatase activity [43]. Tau phosphorylation at multiple sites has been found in mammals that experience hibernation such as arctic ground squirrels, Syrian hamsters and American black bears [44–46]. Increased tau phosphorylation during hibernation is physiologic and likely due to decreased metabolic rate, which can affect both kinase and phosphatase activity [46]. This process is reversible since phosphorylation levels return to normal levels after arousal. Similarly, drug-induced anesthesia can also lead to hypothermia and induce hyperphosphorylation by either activating kinases or by inhibiting phosphatase activity, which returns to normal levels after anesthesia is removed [47, 48].

Site-specific phosphorylation can regulate tau-MT interactions and MT assembly. The MTBR region is generally positively charged to improve interactions with negatively charged MT and the addition of a negative phosphate group via phosphorylation generally results in decreased MT binding. Some studies have shown that tau phosphorylation at Ser231 or Ser262 can directly inhibit MT interactions [49, 50]. Site specific tau phosphorylation of other sites such as Ser208 lead to increased MT binding, suggesting that the regulation of MT binding greatly depends on the phosphorylation site [51]. In addition to MT binding, phosphorylation at Thr231, Ser262, Ser396, or Ser404 can decrease the ability of tau to promote MT assembly and regulate MT dynamic stability [52]. Interestingly, multi-site phosphorylation can restore normal MT assembly and may be inherently complex in its modulation of MT function [52]. The consequences of MT regulation also can affect axonal transport: tau phosphorylation at Tyr18 can regulate tau’s inhibition of motor proteins such as kinesin-1 and is generally a protective mechanism that promotes physiological axonal trafficking [53, 54]. Conversely, phosphorylation of Ser422 can overall inhibit anterograde and retrograde axonal transport [55]. These studies demonstrate that the physiologic MT regulation by tau is bidirectionally modulated by the sum of multiple phosphorylation sites.

### Evidence of elevated tau phosphorylation in AD

Alzheimer’s disease (AD) is the most common form of dementia and a devastating neurodegenerative disorder that affects over 5.8 million Americans [56]. By 2050, this number is projected to increase to 13.8 million [56]. Along with Aβ amyloid plaques, neurofibrillary tangles (NFT) made of phosphorylated tau are one of the major pathological hallmarks of AD and are cornerstones of the current NIA criteria for the post-mortem diagnosis of AD neuropathologic change [57, 58]. NFT directly correlate with progressive decline in dementia scores and development of cognitive deficits, making phosphorylated tau (p-tau) a potential driver of neurotoxicity in AD [59, 60].

**Fig. 2:**
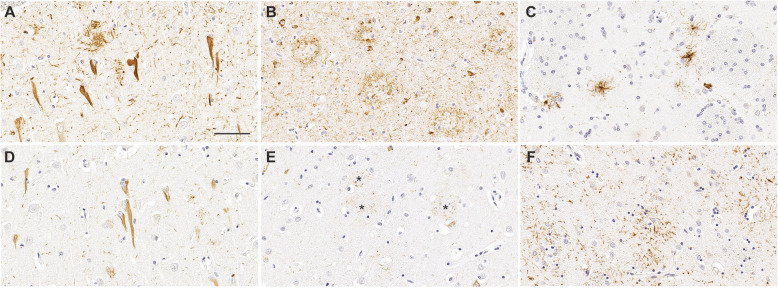
Tau pathological inclusions associated with neurodegenerative diseases stained with antibodies specific for p-tau. Neurofibrillary tangles (**A**) and neuritic plaques (**B**) in AD stained with antibody AT8 that reacts with tau phosphorylated at Ser202 and Thr205. Tufted astrocytes (**C)** in PSP stained with antibody AT8. Antibody 3G12 specific for tau phosphorylated at Ser208 depicts neurofibrillary tangles (**D**) and neuritic plaques (**E**, asterisks) in AD. Astrocytic plaques (**F**) in CBD stained with antibody AT8. Scale bar = 60 μm

In early studies, aggregated tau isolated from AD brains was found to have 3–4 fold higher overall phosphorylation levels (8 mol per protein) compared to healthy controls (2–3 mol per protein) [61]. This increase in tau phosphorylation within pathological inclusions allows for their detection with tau phosphorylation-specific antibodies (Fig. 2). Numerous mass spectrometry studies have mapped phosphorylation sites in detergent-insoluble tau isolated from AD and healthy control brains [1–4]. A recent quantitative mass spectrometry study examined a cohort of 49 AD patients and 42 AD control subjects and greatly expanded the number of potential phosphorylation sites found in AD and control brains [5]. A compilation of these results reveals at least 59 phosphorylation sites found in AD compared to at least 19 sites in control brains (Listed in Table 1 and shown in Fig. 1). There is considerable overlap between many sites found in AD and control brains.

**Table 1:** List of phosphorylation sites found in brain and CSF from AD patients versus healthy controls. Phosphorylation sites found in brains of AD and age-matched controls were based on mass spectrometry studies that isolated PHF-tau [1–5]. Fewer studies have attempted to map phosphorylated tau within the CSF of AD and age-matched controls [62, 63]. *Tyr18 has been identified in immunocytochemical staining, but not detected by mass spectrometry

Phosphorylation Sites	Control Brain	AD Brain	Control CSF	AD CSF [62, 63]
Tyr18	X*	X*		
Tyr29		X		
Thr30		X		
Thr39		X		
Ser46	X	X	X	X
Ser56		X		
Ser61			X	X
Ser68		X		
Thr69		X		
Thr71		X		
Thr102		X		
Thr111		X	X	X
Ser113		X		X
Thr153		X		X
Thr175		X		X
Thr181	X	X	X	X
Ser184	X	X		
Ser185		X		
Ser191		X		
Ser198	X	X		
Ser199	X	X	X	X
Ser202	X	X	X	X
Thr205		X	X	X
Ser208		X	X	X
Ser210		X		
Thr212	X	X		
Ser214	X	X	X	X
Thr217	X	X	X	X
Thr220		X		
Thr231		X	X	X
Ser235	X	X	X	X
Ser237		X		
Ser238		X	X	X
Ser241		X		
Ser258		X	X	X
Ser262	X	X	X	X
Thr263		X		
Ser285			X	
Ser289		X	X	
Ser293		X		
Ser305		X		
Tyr310		X		
Ser316		X		
Ser352		X		
Ser356		X		
Thr361		X		
Thr386		X		
Tyr394		X		
Ser396	X	X	X	X
Ser400	X	X	X	
Thr403	X	X	X	
Ser404	X	X	X	
Ser409		X		
Ser412	X	X		
Ser413	X	X		
Thr414	X	X		
Ser416	X	X		
Ser422		X		
Ser433		X		
Ser435		X		

Since hyperphosphorylation is a major component of tau pathological inclusions, phosphorylation-specific antibodies have been very useful for tracking the progression of tau pathology. Braak staging of NFT pathology in AD was traditionally performed with Gallyas silver stain [64]; however, the AT8 antibody against tau phosphorylated at Ser202/Thr205 was found to be just as effective at tracking the progression of tau pathology [65, 66]. According to the Braak staging scheme, tau spreads in a stereotypical manner in most patients: NFT composed of hyperphosphorylated tau are found in the transentorhinal region in early stages (stage I-II), in limbic regions such as hippocampus in middle stages (stage III-IV), and throughout the neocortex in late stages (stage V-VI) [64, 65]. Phosphorylation at other sites has been found to be distinctively increased in more advanced Braak stages (stages V/VI) including Tyr18, Ser199, Thr231, and Ser422 [67]. Interestingly Tyr18 and Thr231 are found to be phosphorylated in earlier Braak stages (stages III/IV) [67].

In a follow up study, Braak and colleagues also found that some cases had unique types of AT8 positive and Gallyas negative pre-tangles in the brainstem and some cortical areas, even before any bona fide NFT were detectable in the transentorhinal region. These cases were designated as prodromal stages with pre-tangles that contain phosphorylated tau [68]. Antibodies against other phosphorylation sites such as Thr153, Thr231, and Ser262 have stained pre-tangles early in disease [69]. These findings suggest that different phosphorylation sites are distinctively involved in early versus late stages of NFT progression in AD.

### Effects of tau hyperphosphorylation on microtubule interactions, aggregation and prion-like spread

In pathologic states like AD, hyperphosphorylated tau exhibits impaired MT binding and is less capable of promoting MT assembly contributing to a breakdown of MT in late stages of AD [70]. Another consequence of decreased MT binding is the mislocalization of tau from the axon to the cytosol. In a tau knock-in mouse model with 27 different phosphomimetics that model hyperphosphorylation, axonal tau was mislocalized into the somatodendritic compartment [71], similar to what is observed in early tau lesions in human brains.

Multi-site phosphorylation can lead to conformational changes that make tau protein prone to misfolding and aggregation [72]. Experimental tau pseudophosphorylation at Ser199, Ser202, Thr205, Ser396, and Ser404 led to disruption of the paperclip conformation and induction of a pathological conformation that may be more prone to aggregation [73]. Multiple phosphorylation sites such as Thr175, Ser202, Thr205, Thr212, and Ser422 have been shown to promote aggregation and lead to the formation of tau filaments in vitro [55, 74–76]. Triple phosphorylation at Ser202/Thr205/Ser208 has been proposed to be a combination that leads to rapid tau aggregation [51, 77]. On the other hand, phosphorylation sites such as Ser214, Ser262, and Ser305 have been demonstrated to inhibit tau aggregation and may be neuroprotective [78, 79]. In late disease stages, the hyperphosphorylation of multiple pro-aggregation sites may overcome protective ones. It is also unclear whether certain specific tau phospho-epitopes are added before or after the formation of tau inclusions, which may mitigate potential neuroprotective effects.

Like other proteins involved in neurodegeneration, tau has been proposed to spread by prion-like mechanisms where tau seeds can induce aggregation of wild type tau and spread from neuron to neuron [80–82]. Both unmodified tau fibrils [83] and phosphorylated tau seeds [84] can induce the spread of tau pathology when injected into the brains of tau transgenic mice. When these seeds are dephosphorylated by phosphatases, they are less effective at inducing tau pathology. Similarly, tau seeds that were immunodepleted with phosphorylation-specific antibodies were less capable of inducing tau aggregation, suggesting that phosphorylation may be a characteristic of potent tau seeds [85]. Specific phosphorylated tau residues including Ser262, and combined Thr231/Ser235 found in AD brain lysate enhance tau seeding efficiency, while other sites such as Ser198/Ser199/Ser202 and Ser400/Thr403/Ser404 can be associated with lower seeding efficiency [86]. This suggests that tau phosphorylation can heterogeneously modulate tau seeding capacity by either enhancing or diminishing tau spread.

### Heterogeneity of tau species in different Tauopathies

In addition to AD, tauopathies encompass a group of heterogeneous neurological diseases that share the common feature of brain-related tau pathological inclusions. These include subtypes of Frontotemporal lobar degeneration (FTLD)-tau, such as corticobasal degeneration (CBD), progressive supranuclear palsy (PSP), Pick’s Disease (PiD), globular glial tauopathy (GGT) and argyrophilic brain disease (AGD), as well as the distinct entity of chronic traumatic encephalopathy (CTE). Various missense, silent and intronic *MAPT* mutations cause familial forms of frontotemporal dementia with parkinsonism [87, 88] and these mutations are associated with tau hyperphosphorylation, MT dysfunction, and aggregation [87, 89–92]. Intronic, silent and some of the missense mutations cause disease by altering the splicing efficiency of tau exon 10 and changing the ratio of 3R to 4R isoforms [93]. Some *MAPT* missense mutations reduce MT binding and increase phosphorylation, while a small subset of them promote tau aggregation [87, 89–92]. Cases with *MAPT* mutations have traditionally been referred to as frontotemporal degeneration and parkinsonism linked to chromosome 17 (FTDP)-17, but recent nomenclature schemes re-classify them under familial forms of FTLD-tau since patients with familial tau mutations have similar tau pathology as sporadic FTLD cases [94] and to avoid confusion with cases of FTLD-TDP associated with mutations in the GRN gene which is also found on chromosome 17.

Within the group of tauopathies, there is diversity in the morphology, regional distribution and cell type specificity of tau inclusions [95]. Several of the major tauopathies including AD, CBD, and PSP are shown in Fig. 2 with staining by AT8 (pSer202, pThr205) or 3G12 (pSer208) antibodies. In AD, tau pathology consists mostly of neuronal pathology including NFT (Fig. 2A, D), neuropil threads, and dystrophic neurites within senile plaques (Fig. 2B, E). In addition to neuronal tau pathology in the form of globose tangles, subtypes of FTLD-tau are associated with glial tau inclusions including tufted astrocytes in PSP (Fig. 2C) and astrocytic plaques in CBD (Fig. 2F) [96]. PiD has distinctive cytoplasmic Pick bodies in neurons [97], while GGT has characteristic globular glial inclusions [98]. CTE, a secondary tauopathy associated with repetitive brain injuries, develops pathognomonic p-tau positive inclusions in neurons, astrocytes and cell processes mostly around blood vessels and distinct cortical regions [99].

The differences in tau pathology are also reflected structurally within different tau filament folds. Recent cryo-electron microscopy studies have uncovered differential tau structure for AD, PiD, CTE, and CBD [100–103]. PTMs may partially contribute to conformational differences of tau filaments. Ubiquitination and acetylation of lysine sites may differ between AD and CBD tau filaments [104]. Different phosphorylation sites may also help differentiate AD from other tauopathies. For example, pSer208 antibody which strongly stained neuronal NFT, but barely detected astrocytic pathology in PSP and CBD [51]. This may be due to the different kinase environment in various forms of tau inclusions.

Due to the structural and PTM differences, tau filaments from different tauopathies may represent separate tau prion-like strains. Human brain lysates from AD, PSP, CBD, AGD and GGT induce different forms of tau pathology when injected into the brains of tau transgenic mice and even non-transgenic mice [105–108]. AD brain lysate results in primarily neuronal pathology, while PSP, CBD, AGD and GGT lead to disease-specific glial pathology. Since tauopathies may represent different tau strains, there is potential for biomarkers like phosphorylation sites that could differentiate AD from other tauopathies.

### Development of p-tau CSF and plasma biomarkers

The National Institute on Aging and Alzheimer’s Association recommended defining AD by neuropathology and biomarkers for tracking of disease progression [109]. Biomarkers have been grouped in the ATN framework as either detecting Aβ aggregation (A), tau aggregation  (T), or neurodegeneration (N). Currently, the gold standards for tau-based biomarkers include pThr181 tau within the cerebrospinal fluid (CSF) and tau detected by positron emission tomography (PET) [109]. Multiple studies are ongoing to evaluate different tau-specific PET tracers [110]. However, there is a need for the expansion of multiple biomarkers. Here, we present an overview of recent developments in novel biomarker assays for the detection of different p-tau sites in CSF and blood.

### CSF p-tau biomarkers

CSF is produced by ependymal cells of the choroid plexus and can be sampled by lumbar puncture. The development of an effective CSF tau biomarker could help track the progression of AD and symptoms. Overall tau protein levels and p-tau are significantly elevated in AD compared to controls. Much of the tau proteins in CSF are truncated tau fragments that are often phosphorylated [62, 63]. Most of the phosphorylation sites within the CSF are clustered in the proline rich domain, which suggests a unique set of kinases that are responsible for phosphorylation of tau secreted into CSF. Table 1 summarizes at least 19 major phosphorylation sites that are significantly elevated in the CSF of AD compared to healthy adults.

Soluble phosphorylated tau in CSF has been identified to be a potential early marker of AD disease progression. Increased pThr217 and pThr181 appear simultaneously during the formation of Aβ amyloid plaques, about 20 years before the onset of AD [111]. pThr205 was found to be elevated later when decreased brain metabolism and neuronal dysfunction occur about 13 years before AD onset. In the final stages, NFT formation is detected by PET-tau imaging during AD onset. These findings show that soluble hyperphosphorylated tau species are distinctively elevated within the CSF decades before AD diagnosis.

#### CSF pThr181 tau

From a meta-analysis of p-tau CSF studies in AD, pThr181 is one of the most evaluated CSF tau biomarkers and is consistently elevated in AD compared to age-matched healthy controls [112, 113]. It is considered one of the gold standards and is included within the biomarker definition of AD [109]. pThr181 is also elevated in patients with mild cognitive impairment (MCI) [114]. Interestingly, pThr181 is increased in AD, but in non-AD dementia such as FTD, pThr181 is significantly decreased compared to healthy controls [115, 116]. In a cohort of 361 participants, a low ratio of pThr181 to total tau levels can differentiate FTD from healthy controls with 73% sensitivity and 93% specificity [115]. However, pThr181 is not significantly different between PSP, CBD, or other FTD variants [115].

#### CSF pThr217 tau

Since pThr217 was found to be elevated early along with pThr181, several new immunoassays have been developed to directly compare pThr217 with pThr181. Eli Lilly developed a new ELISA to detect pThr217. In AD, pThr217 had a higher range of 7.3–9.6 fold change versus 3.6–3.7 fold change in pThr181 [117]. Compared to Thr181, pThr217 correlated better with progressive NFT pathology as determined by PET imaging. pThr217 was slightly better at differentiating AD from non-AD with a 91% sensitivity and 91% specificity, compared to pThr181 which had 79% sensitivity and 96% specificity [117].

Another ELISA assay detects pThr217 using validated antibodies, DC2E2 and DC2E7, that both detect tau pathology in AD and other tauopathies [118]. In a direct comparison, they showed that pThr217 can distinguish AD from healthy controls with 98% specificity and 93% sensitivity [118]. This is slightly better than pThr181 that identified AD with specificity and 86% sensitivity. pThr217 was also better at differentiating AD from tauopathies like PiD, PSP, and CBD with a 90% accuracy, while pThr181 had lower accuracy of 78% [118].

A different approach is to use quantitative mass spectrometry to determine p-tau changes in CSF [119]. With this method, pThr217 had 6-fold change in AD patients compared controls, while pThr181 was elevated with 1.3-fold change. Overall levels of pThr217 may be higher than pThr181, which could make it easier to detect by immunoassays.

#### CSF pThr231 tau

Another early epitope is pThr231, which has been detected to be elevated in CSF of patients with AD and patients with MCI when compared to healthy controls [120, 121]. pThr231 can differentiate patients with AD from other conditions such as FTD, vascular dementia, and LBD with 90.2% sensitivity and 80% specificity [122]. For identifying patients with AD, pThr231 may have better sensitivity than pThr181 and detect less false positives [123]. Interestingly, AD patients who are ApoE ε4 carriers have higher levels of pThr231, but no difference in pThr181 [123, 124]. Another study found that pThr231, pThr181, and pThr217 were similarly elevated in preclinical AD, before the appearance significant Aβ pathology [125]. All three of these phosphorylation sites are likely elevated during early stages of AD and can be detected in the CSF.

Many of the recent studies have been designed to determine the best biomarker for AD and other tauopathies. However, it can be difficult to compare the results of different studies due to the variety of antibodies used in different immunoassays. The choice of detection and capture antibody in sandwich ELISAs greatly affects the efficacy of these assays. For instance, many immunoassays use similar antibodies against pThr181 and pThr217 as the capture antibody but may differ in the choice of the detection antibody. Instead of using the standard mid-domain tau antibody for detection (amino acid residues 159–163), another group used an N-terminal tau antibody (amino acid residues 6–18) as the detection antibody to bind a longer tau peptide. The N-terminal directed pThr181 and pThr217 immunoassays were better than the mid-domain directed pThr181 assay at differentiating patients with AD and MCI [126]. There are many additional p-tau sites found within the CSF (Table 1) that could be new potential sites that predict different stages of AD progression. There may also be phosphorylation signatures specific to other tauopathies that are not found in AD. In the future, expanding the repertoire of p-tau biomarkers and using a combination of multiple assays will improve the diagnostic accuracy and tracking of AD and other tauopathies.

#### Plasma p-tau biomarkers

Blood-based p-tau biomarkers are currently in development and evaluated for their prognostic value primarily in AD. Blood tests are advantageous over CSF tests, because they are minimally invasive and less expensive to evaluate in a large population. Since there are lower levels of tau protein in the blood compared to CSF, blood tests may require a highly sensitive assay to detect minute amounts of p-tau. Other cell types and proteins found in blood may further complicate detection. Overall plasma tau levels are elevated in AD compared to patients with MCI and healthy controls [127]. Due to the success of p-tau in CSF, p-tau epitopes such as pThr181 and pThr217 were also evaluated in plasma ELISA assays.

#### Plasma pThr181 tau

Plasma pThr181 levels are significantly elevated in AD at 3.5 times more compared to healthy controls [128]. It generally correlates with CSF pTau181 levels and Aβ levels measured by PET imaging [128, 129]. Additionally, Plasma pThr181 has diagnostic value and can differentiate AD from other neurodegenerative diseases such as FTLD with 70–80% sensitivity and specificity [128]. A longitudinal prospective study tracked a cohort of 1113 participants with follow up 8 years later and found that plasma pThr181 tau levels is associated with progressive decline in cognitive symptoms and brain atrophy [130]. This association was also found in patients with MCI, suggesting that plasma pThr181 could be an early predictor of neurodegeneration and cognitive decline.

#### Plasma pThr217 tau

Similar to plasma pThr181, Plasma pThr217 is increased in AD and can distinguish AD from other neurodegenerative diseases such as PD, PSP, and FTD in a cohort of 1402 participants [131]. Comparable to CSF pThr217 assays, plasma levels were also associated with worsening cognitive scores and overall brain atrophy [131, 132]. Plasma pThr217 may be a good predictor of early disease progression prior to AD. In a cohort of 490 participants of healthy controls and patients with MCI, both plasma and CSF pThr217 tau increased before major changes on tau PET imaging [132]. As an early marker, plasma pThr217 can predict the progression of AD in a longitudinal study tracked a cohort of 250 participants up to 6 years. Patients with MCI who eventually developed AD had higher baseline pThr217.

Recent advancements in plasma p-tau assays have demonstrated that these can be as effective as their CSF counterparts. Both plasma pThr181 and pThr217 tau are good early biomarkers of AD and predictor of disease progression in multiple longitudinal studies. Plasma p-tau is highly elevated in AD compared to other related neurodegenerative diseases such as FTD. Further studies with larger sample sizes will better validate the efficacy of plasma p-tau levels compared to other markers in AD. A combination of multiple p-tau biomarkers in addition to pThr181 and pThr217 could improve the sensitivity and specificity of tracking early AD progression.

### Therapies that target tau phosphorylation

**Fig. 3:**
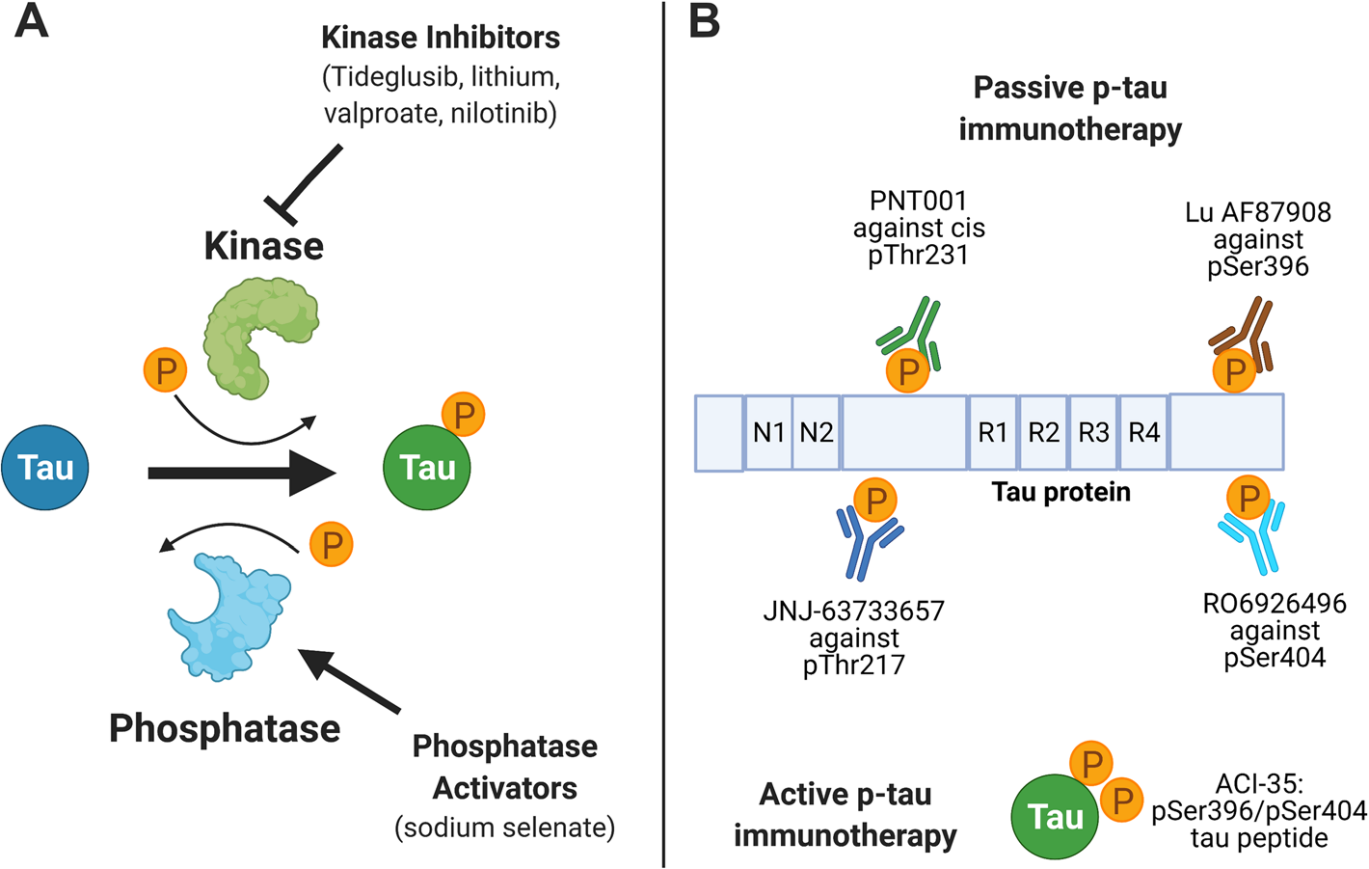
Summary of therapies targeting p-tau. **A** Kinase inhibitors such as Tideglusib, lithium, valproate and nilotinib act to prevent hyperphosphorylation. Phosphatase activators such as sodium selenate increases dephosphorylation activity. **B** Passive p-tau immunotherapy are specific antibodies that target p-tau epitopes for degradation. Active p-tau immunotherapy involves immunization with a p-tau peptide to generate antibodies. Figure was made with Biorender

Currently, there is a lack of effective disease-modifying treatments for AD. Tau and particularly tau phosphorylation, has become an attractive target, because it is involved in early disease progression and can be tracked by various biomarkers. Recently, several clinical trials primarily in Phase I/II have been completed or ongoing involving several therapeutic strategies to reduce tau phosphorylation: kinase inhibitors, phosphatase activators, and p-tau immunotherapy (Fig. 3). Kinase inhibitors and phosphatase activators aim to decrease tau phosphorylation levels early on (Table 2) and p-tau immunotherapy will lead to the clearance of phosphorylated tau (Table 3), thus achieving a similar goal.

**Table 2:** List of clinical studies on tau kinase inhibitors and phosphatase activators

Drug Class	Drug Name	Mechanism	Phase	Clinical Trial ID	Population	Status
Kinase Inhibitor	Tideglusib	Inhibits GSK3-β	Phase II	NCT01350362	Mild AD	Completed
			Phase II	NCT01049399	Mild PSP	Completed
			Phase I/II	NCT00948259	AD	Completed
	Lithium	Inhibits GSK3-β	Phase II	ISRCTN72046462	Mild AD	Completed
			Phase II	NCT00088387	AD	Completed
			Phase II	NCT01055392	MCI	Completed
			Phase I/II/III	NCT02601859	MCI	Completed
			Phase IV	NCT03185208	MCI	In Progress until 2023
	Valproate	Inhibits GSK3-β	Phase I	NCT01729598	Healthy adults	Completed
			Phase II	NCT00088387	AD	Completed
			Phase III	NCT00071721	AD	Completed
	Nilotinib	Inhibits tyrosine kinase	Phase II	NCT02947893	mild/moderate AD	Completed
Phosphatase Activator	Sodium Selenate	Activates PP2A	Phase II	ACTRN 12611001200976	Mild/moderate AD	Completed
			Phase II	ACTRN 12620000236998	Behavioral variant of FTD	In Progress until 2024

**Table 3:** List of p-tau specific immunotherapy drugs currently in clinical development

Drug Name	Mechanism	Phase	Clinical Trial ID	Population	Status
JNJ-63733657	Antibody against pThr217	Phase I	NCT03375697	Healthy Adults	Completed
		Phase I	NCT03689153	AD, Healthy Adults	Completed
		Phase II	NCT04619420	AD	In Progress until 2025
PNT001	Antibody against Cis isomer of pThr231	Phase I	NCT04677829	Patients with Traumatic Brain Injury	In Progress until 2022
		Phase I	NCT04096287	Healthy Adults	In Progress until 2021
Lu AF87908	Antibody against pSer396	Phase I	NCT04149860	AD	In Progress until 2021
RO6926496	Antibody against pSer404	Phase I	NCT02281786	Healthy Adults	Completed
ACI-35	Vaccine of tau peptide with pSer396/pSer404	Phase I/II	NCT04445831	AD	In Progress until 2023

### Kinase inhibitors and phosphatase activators

#### Tideglusib

Tideglusib is a thiadiazolidinone-based drug that can competitively inhibit the enzymatic activity of glycogen synthase kinase 3-β (GSK3-β) [133, 134]. In transgenic mouse models that express both tau and Aβ pathology, Tideglusib reduced tau phosphorylation and Aβ levels, ameliorated neuronal death, and rescued cognitive memory deficits [135].

Due to these positive preclinical results, a pilot Phase I trial of Tideglusib tested safety and different doses (400, 600, 800, and 1000 mg) given daily for 4–6 weeks in patients with mild or moderate AD [136]. There were no major side effects except for elevated serum transaminases found on liver function tests. Patients on the 1000 mg daily dose schedule showed an uptrend in dementia scores, but due to the small sample size of 30 participants, it may be difficult to extrapolate the results.

A follow-up Phase II study expanded the number of participants to 306 patients with mild or moderate AD, testing 500 mg versus 1000 mg daily doses [137]. Similar to the previous study, there were no major side effects found although 14–18% of patients experienced diarrhea, and a few had transaminase elevations. Overall, the results showed that there was no significant clinical benefit as determined by the primary outcome of dementia inventory scores. There was a slight uptrend for the 500 mg dose group with improvements in different dementia scores and word fluency.

Another phase II trial tested the effects of Tideglusib in 37 patients with PSP at 600 or 800 mg daily for a year [138]. Tideglusib treatment reduced brain atrophy throughout the brain, especially for the parietal and occipital lobes as measured by MRI imaging. There were no major changes in clinical dementia scores, but the reduction of brain atrophy is very promising and the extension of treatment time to 1 year may have a stronger effect.

#### Lithium

Lithium salts are oral drugs approved for treatment of mood disorders including bipolar disorder and major depressive disorder [139]. Lithium was initially mainly considered for the treatment of agitation and psychosis in AD [140]. Preclinical studies suggest that lithium is also a direct reversible inhibitor of GSK-3β activity and may be capable of decreasing tau hyperphosphorylation and slowing AD disease progression [141]. In transgenic mouse models, chronic treatment with lithium prevented tau phosphorylation and slowed disease progression [142, 143], but existing mature NFT were not significantly removed [142].

In an initial trial of 71 mild AD patients, 10 weeks of lithium (titrated to a maximum of 336 mg daily dose) did not significantly alter CSF p-tau or lymphocyte GSK-3β activity [144]. The other primary outcomes of dementia scores were also not changed. Subsequent trials had more success with longer treatment periods and in patients with early MCI. Forty-five patients with MCI received 150 to 600 mg daily doses for 1 year, which significantly decreased CSF pThr181 tau and improved cognitive symptoms as measured by the clinical dementia rating (CDR) [145]. Similar results were positive in another trial of 61 patients with MCI that received lithium for 2 years [146]. Overall, they received more stable scores on Alzheimer’s disease assessment scale (ADAS) and CDR dementia scales. Even lower doses at 300 μg daily for 15 months in AD patients was able to prevent declining MMSE scores [147]. Longer treatment schedules and use in patients with early symptoms like MCI showed promising results for biomarker and dementia score improvements. A current phase IV clinical trial of lithium in MCI patients is ongoing until 2023 [148].

#### Valproate

Valproate is approved for treatment of bipolar disorder and epilepsy [149, 150]. In the context of tau, valproate has been demonstrated to be an inhibitor of both GSK3-β and cyclin dependent kinase 5 (cdk5), thus decreasing tau phosphorylation [151]. This was confirmed in transgenic mouse models where valproate beneficially restored synaptic loss and decreased tau phosphorylation [152]. Another study found that valproate inhibits Aβ plaque formation and improved cognitive deficits [153].

Initially, valproate was considered for clinical use of treating agitation and aggression in AD [154, 155], but it could also potentially slow AD progression by targeting tau and Aβ pathology. A phase II trial found that AD disease progression and cognitive decline was not significantly altered by valproate treatment [156]. Another phase II trial showed that valproate led to worsening dementia scores as assessed by the mini-mental state examination (MMSE) [157]. Both studies detected that valproate treatment resulted in increased brain atrophy on MRI imaging [156, 157]. This is a rare side effect of valproate that leads to reversible brain atrophy and has been previously reported [158, 159]. Due to the serious side effects and lack of efficacy, valproate may not be the best candidate compared to other kinase inhibitors.

#### Nilotinib

Nilotinib is a pyrimidinyl benzamide inhibitor of the tyrosine kinase Abl and is primarily used to treat chronic myeloid leukemia [160]. Recently, Nilotinib has been considered for use in neurological disease such as AD. Abl tyrosine kinase is involved in tau phosphorylation and its inhibition could slow hyperphosphorylation. Nilotinib showed other benefits such as autophagic clearance of phosphorylated tau and improvements in neurotransmitter imbalance in tau transgenic mice [161]. Nilotinib may also help clear Aβ amyloid and improve cognitive deficits by affecting soluble parkin [162].

A phase II trial tested safety and tolerance of nilotinib in 37 patients with mild or moderate AD and measured different biomarkers [163]. Nilotinib was given daily for 26 weeks in either 150 or 300 mg doses. These treatments were generally well tolerated with no significant side effects except for mood swings with agitation and irritability in the higher dosed 300 mg group. Measurement of biomarkers showed reduction of pThr181 tau and Aβ in CSF and slowing of hippocampal atrophy as measured by imaging. These results support the potential benefit of nilotinib on reducing both tau and Aβ pathology.

#### Sodium selenate

Sodium selenate has been identified as a potent activator of PP2A phosphatase enzymatic activity, leading to tau dephosphorylation [164, 165]. In tau transgenic mouse models, oral treatment with sodium selenate reduces tau hyperphosphorylation, tau pathology progression, and behavioral and cognitive deficits [164–166]. By targeting tau phosphorylation in the early stages, it could be possible to slow or prevent the formation of tau inclusions.

In Australia, a phase II clinical trial examined supranutritional levels of VEL015, a formulation of sodium selenate (10 mg taken three times daily), for 24 weeks in patients with mild or moderate AD [167]. Sodium selenate was generally safe with some minor side effects of headache, fatigue and nausea. There were no major changes in the dementia scores, but there was a significant improvement in MRI diffusivity, indicating increased membrane integrity. The exploratory measurement of total tau and pThr181 tau in CSF showed no major differences, but there was a downward trend.

A follow-up study collected data from the previous trial and measured serum and CSF selenium levels, showing that selenate uptake into CSF varied greatly from patient to patient [168]. In a group with high CSF selenium levels, these patients showed improvement in MMSE cognitive scores. Future studies that better control CSF selenate uptake could show better improvement in dementia scores and changes in CSF tau biomarkers. A new phase II trial on sodium selenate in behavioral variant of FTD will use higher doses (15 mg taken three times daily) in a target cohort of 120 patients [169]. This trial is currently in recruitment and is expected to be completed by 2024.

Overall, kinase inhibitors and phosphatase activators have shown some effect at altering tau biomarkers, but effects on cognitive symptoms were mixed. Some kinase inhibitors like valproate have serious side effects. Lower doses and slower titration schedules could help improve tolerance and decrease complications. Targeting kinase and phosphatase activity likely will likely have a larger clinical effect during early disease, such as in patients with MCI or mild AD.

### Phospho-tau immunotherapy

Tau immunotherapy is one of the most promising tau-targeting therapeutics in recent years due to much greater target specificity. There are two major strategies: the first is active immunotherapy which involves the injection of a peptide to generate an intrinsic immune response against that epitope. An advantage is that multiple antibodies may arise from a given vaccine and offer sustained protection, but this may not be as effective in older patients with weaker immune systems. The second strategy is the use of passive immunotherapy or the peripheral infusion of an antibody that can bind a specific epitope, but this would require repeat infusions. Passive immunotherapy has proved to be more popular, since preclinical studies in different animal models have demonstrated that tau-targeting antibodies can lead to clearance of toxic tau aggregates and inclusions [85, 170–174]. This strategy also allows an active pipeline for clinical development since an antibody made in other mammals such as mice or rabbits can be screened and humanized in a cost-efficient way. Humanized antibodies retain its variable binding domain and epitope specificity, but its constant domain is human to reduce unwanted immune responses to the antibody. In addition to tau antibodies, tau intrabodies to target intracellular tau have also been effective in preclinical studies, but have not been tested in clinical trials [175, 176].

Peripheral administration such as intravenous routes may only deliver small amounts of antibodies to the central nervous system across the blood brain barrier (BBB). In MCI and dementia, the BBB can be slightly leaky [177, 178] allowing for higher levels of antibodies to enter the CNS [170, 179]. Tau-targeting antibodies may be taken up by neurons via clathrin-dependent endocytosis and lead to autophagy-dependent protein degradation of tau aggregates [180]. Extracellularly, antibodies can bind to secreted tau and activate microglia to engulf and help degrade tau aggregates based on cell culture experiments [181]. This could inhibit transcellular spread of tau from neuron to neuron, thus slowing the disease progression of different tauopathies [85, 182].

The efficacy of passive immunotherapy will largely depend on choice of epitope and properties of the antibody such as binding affinity. Many antibodies target total tau and aim to lower overall tau levels, but this may also decrease physiologic functional tau. The alternative is to target phosphorylation-specific epitopes, which can allow for more specific clearance of pathogenic tau with less removal of functional tau that may be needed to maintain MT and axonal transport. However, specific phospho-tau antibodies are unlikely to capture all tau species. In recent years, many antibodies tested in preclinical models have been humanized and taken to phase I and phase II clinical trials. Many of these immunotherapies target phosphorylation epitopes in the proline rich region or N-terminus of tau. Here, we will summarize the current progress of p-tau specific immunotherapy and ongoing clinical trials (listed in Table 3).

#### JNJ-63733657 antibody against pThr217 tau

JNJ-63733657 is a humanized IgG1 antibody that is specific for tau phosphorylated at Thr217 and is under development by Janssen Biotech, a subsidiary of Johnson and Johnson. It was found that targeting phosphorylated tau epitopes near the proline-rich domain was effective at blocking the spread of tau seeds in cell-based assays and in animal models [183]. Based on these positive results, the antibody was humanized and tested in two Phase I trials [184, 185]. In the initial Phase I trial, healthy subjects from 55 to 75 years of age were treated with intravenous injections of JNJ-63733657 [186, 187]. The antibody was generally well tolerated without significant side effects, though some patients experienced back pain and headache. Antibody concentrations increased linearly with the dosage amount and can enter the CSF. Based on these positive results, a Phase II trial is started in 2021 to recruit up to 420 participants with early AD to test a low and high dose injection every 4 weeks for several years [188]. The trial is expected to conclude in 2025.

#### PNT001 against cis p-Thr231 tau

Tau phosphorylation at Thr231 can exist in either a cis or trans isomer and its interconversion is catalyzed by Pin1, a propyl isomerase [189]. Cis-pThr231 tau but not trans-pThr231 tau impairs MT assembly and is prone to tau aggregation in AD and CTE [172, 190]. A monoclonal cis-pThr231 tau antibody (clone 113) was developed to be cis isomer specific at pThr231 [171]. In mouse models of traumatic brain injury (TBI), peripheral delivery of cis- pThr231 antibody prevented short term and long-term outcomes of TBI, leading to reduced spread of tau axonal pathology, decreased neuroinflammation, and improved motor coordination deficits [171, 172]. On the other hand, the trans-pThr231 tau antibody was not effective, suggesting that cis-pThr231 tau is the primary driver of tau pathology and neurodegeneration in TBI. The cis-pThr231 tau antibody was humanized as PNT001 by Pinteon Therapeutics and will evaluated in ongoing Phase I trials of healthy adults and patient with acute TBI, which are scheduled to be completed in 2021 and 2022 [191, 192].

#### Lu AF87908 antibody against pSer396 tau

To help inhibit cell-to-cell spread of hyperphosphorylated tau, C10.2 antibody was screened as a mouse monoclonal antibody that can specifically target tau phosphorylated at Ser396. Tau seeds immunodepleted by C10.2 are less competent at inducing tau aggregation and spread in cellular assays and in rTg4510 tau transgenic mice [85]. Specifically, the C10.2 antibody has been demonstrated to help mediate lysosomal degradation of pathogenic tau by microglia in cell culture experiments [181], and in general, antibody uptake is dependent on its isotype and other characteristics [193]. This antibody has been humanized as Lu AF87908 by H. Lundbeck company and will be tested in a Phase I clinical trial, which is expected to be completed in May of 2021 [194].

#### RG7345 antibody against pSer422 tau

RG7345 is the humanized form of the Mab86 antibody and is thought to be specific for tau phosphorylated Ser422. Mab86 was made by immunizing rabbits with a tau peptide from 416 to 430 residues that contains phosphorylated Ser422. Peripheral administration of the antibody in triple transgenic mice with both tau and Aβ pathology resulted in significant reduction of tau inclusions [170]. The antibody was confirmed to enter neurons and lysosomes to help clear p-tau. Roche started a Phase I trial on RG7345 to compare the safety profile and serum levels; however, the use of RG7345 has been discontinued and no further trials have been announced [195].

#### ACI-35 vaccine

AC Immune SA developed ACI-35 as a liposome-based vaccine that consists of a C-terminal tau fragment (393–408 residues) containing phosphorylated Ser396 and Ser404. In C57/BL6 and tau-P301L transgenic mice [196], injection of the ACI-35 peptide generated a robust immune response, yielding multiple antibodies that are specific for p-tau and react with tau inclusions [197]. A preliminary Phase I trial of ACI-35 in AD patients showed no major safety concerns, but the vaccine elicited a weak immune response [198, 199]. A new improved version of ACI-35.030 was made with additional epitopes to improve T cell responses, which was tested and confirmed in rhesus monkeys. A Phase I and II trial in patients with early AD is ongoing to determine the safety and immune response of ACI-35.030 based on three different doses [200]. The trial is expected to complete by 2022.

Overall, initial clinical results from p-tau specific immunotherapies have been promising and have a good safety profile without significant side effects. Many of these Phase I and II trials are ongoing in the next few years and these results will be important for future development of other immunotherapies. It will be prudent to develop tau immunotherapies against multiple tau epitopes. There is considerable heterogeneity in the degree of post-translational modifications such as phosphorylation in different patient populations. Different tauopathies may also harbor various tau strains that have variations in phosphorylation signature. The development of a cocktail of p-tau immunotherapies may be the most effective method for efficient removal of pathogenic tau species.

## Conclusion

P-tau is a hallmark of tau pathological inclusions in AD and other tauopathies. During late disease stages, tau aggregates isolated from AD brains are hyperphosphorylated at multiple residues (Fig. 1 and Table 1). Tau hyperphosphorylation can lead to MT dysfunction, mislocalization, and increased propensity for aggregation. However, it is still not completely understood how and if tau hyperphosphorylation directly leads to aggregation and neuronal death. Nonetheless, specific sites are phosphorylated in early disease stages and can provide insight as potential biomarkers. Recent advancements in the development of blood and plasma biomarkers including pThr181 and pThr217 have been good predictors of disease progression from MCI to symptomatic AD and may help differentiate AD from FTD. These non-invasive tests combined with imaging and clinical dementia scores will improve early detection and tracking of potential AD, FTD, and other tauopathies.

Tau phosphorylation is also a potential target for clinical therapies. Several drug classes are currently considered for clinical trials: kinase inhibitors, phosphatase activators, and p-tau immunotherapy (Fig. 3). Kinase inhibitors and phosphatase activators are promising and may work better in patients with MCI or early AD to prevent or slow progression, but are more likely to have off-target effects. Likely, a combination of multiple drugs will be necessary to prevent tau phosphorylation since many kinases are involved and all phosphorylate multiple sites. Many antibodies used for p-tau immunotherapy are still in Phase I and II but have fewer side effects and are relatively safer compared to kinase inhibitors. In the next few decades, more clinical trial data will offer a better picture on whether drugs against p-tau will have disease-modifying effects in AD and other tauopathies.

## Data Availability

Not applicable.

## Abbreviations

Aβ: Beta-amyloid
AD: Alzheimer’s disease
ADAS: Alzheimer’s disease assessment score
AGD: Argyrophilic grain disease
CBD: Corticobasal degeneration
CDK5: Cyclin dependent kinase 5
CDR: Clinical dementia rating
CSF: Cerebrospinal fluid
CTE: Chronic traumatic encephalopathy
FTDP-17: Frontotemporal degeneration and parkinsonism linked to chromosome 17
FTLD: Frontotemporal lobar degeneration
GGT: Globular glial tauopathy
GSK-3β: Glycogen synthase kinase 3β
MAP: Microtubule-associated protein
MAPT: Microtubule-associated protein tau
MARK: Microtubule affinity regulated kinases
MCI: Mild cognitive impairment
MT: Microtubule
MTBD: Microtubule binding domain
NFTs: Neurofibrillary tangles
PET: Positron emission tomography
PHFs: Paired helical filaments
PiD: Pick’s disease
PP2A: Protein phosphatase 2A
PSP: Progressive supranuclear palsy
p-tau: Phosphorylated tau
TBI: Traumatic brain injury
TTBK: Tau-tubulin kinase
